# Statistical Tools for Air Pollution Assessment: Multivariate and Spatial Analysis Studies in the Madrid Region

**DOI:** 10.1155/2019/9753927

**Published:** 2019-02-10

**Authors:** David Núñez-Alonso, Luis Vicente Pérez-Arribas, Sadia Manzoor, Jorge O. Cáceres

**Affiliations:** Laser-Chemical-Group, Department of Analytical Chemistry, Faculty of Chemical Sciences, Complutense University, 28040 Madrid, Spain

## Abstract

The present work reports the distribution of pollutants in the Madrid city and province from 22 monitoring stations during 2010 to 2017. Statistical tools were used to interpret and model air pollution data. The data include the annual average concentrations of nitrogen oxides, ozone, and particulate matter (PM_10_), collected in Madrid and its suburbs, which is one of the largest metropolitan places in Europe, and its air quality has not been studied sufficiently. A mapping of the distribution of these pollutants was done, in order to reveal the relationship between them and also with the demography of the region. The multivariate analysis employing correlation analysis, principal component analysis (PCA), and cluster analysis (CA) resulted in establishing a correlation between different pollutants. The results obtained allowed classification of different monitoring stations on the basis of each of the four pollutants, revealing information about their sources and mechanisms, visualizing their spatial distribution, and monitoring their levels according to the average annual limits established in the legislation. The elaboration of contour maps by the geostatistical method, ordinary kriging, also supported the interpretation derived from the multivariate analysis demonstrating the levels of NO_2_ exceeding the annual limit in the centre, south, and east of the Madrid province.

## 1. Introduction

During the last year, urban air pollution concentrations have increased globally. According to the World Health Organization (WHO), this increase can be estimated at 8% from 2008 to 2013 and more than 80% of people living in urban areas, where air pollution is monitored, are exposed to levels that exceed the limits given by WHO [1]. Urban air pollution is a serious environmental problem, and as urban air quality declines, the risk of stroke, heart diseases, lung cancer, and chronic and acute respiratory diseases, including asthma, increases. In addition, it contributes to damaging building materials and cultural objects [2]. Harmful effects of air pollution and its causes are widely studied [3–5] and, the urban quality declines are mainly related to the increase in traffic emissions, transport-related emissions being the main component of air pollution. A wide variety of air pollutants are emitted by vehicles with petrol-derivatives engines being the most important of them; nitrogen oxides, carbon monoxide, volatile organic compounds (VOCs), and particulate matter have an important impact on air quality in the urban areas [6–10]. Air pollution in big cities and close to the main roadways is dominated by road traffic but the pollution levels are very variable because air pollution is severely influenced by multiple environmental or meteorological factors as well as traffic patterns, size, and orientation of buildings or land use [11–13]. Consequently, determining population exposures is essential to study and understand the causes of these variations prior to the development of interventions and policy recommendation aiming at reduction exposures. In this sense, multivariate statistical techniques are an excellent tool to discover and analyse large dataset of environmental data. There are different methods of dealing with this extensive amount of data, being one of the most interesting to treat all data by means of the application of multivariate analysis methods (i.e., principal component analysis or cluster analysis). The main objective is aimed at grouping and classification of objects (in this case, measured parameters, stations, days, etc.), as well as modelling relationships between the different environmental data. The methods of multidimensional analysis have made it possible to establish some correlations between different parameters and at the same time finding correlations between the amounts of several pollutants [14].

Many multivariate methods can be used in environmental studies because they provide information about association, interpretation, and modelling from large environmental datasets. Correlation analysis is a very useful statistical tool to identify the relationship between pollutants or other variables that affect air quality, and it is very useful to understand or look for the most influential factors or sources of chemical components [15, 16]. This statistical tool has been applied in several studies on air pollution in Chicago [17], performs isotopic analyses in topsoil [16], and identifies sources and correlations between PAHs and heavy metals in Switzerland and Spain [18] and in the urban road dust of Xi'an (China) [19].

Principal component analysis (PCA) like many of the multivariate methods of analysis is based on data reduction, taking into account the correlation between the data. This is possible because only a small number of parameters are significant in a dataset [20]. It has been used extensively in environmental analysis because this statistical analysis method proves to be a very useful aid in data interpretation and classification [1, 21]. Specifically, PCA has been used together with other multivariate techniques, such as canonical correlation analysis (CCA) to uncover existing relationships between meteorology and air pollutants concentrations [17], or with cluster analysis [9, 19, 22–24]. The aforementioned cluster analysis, or more correctly hierarchical cluster analysis (CA), is a sorting method used to divide the data in clusters. With this method, the objects are aggregated stepwise according to the similarity of their features. As a result, hierarchically or nonhierarchically ordered clusters are formed. The ideal number of clusters may be determined graphically through a dendrogram [25]. In general, it can be said that CA is a useful procedure for simplifying and classifying the behaviour of environmental pollutants in a specific region [26–28].

Another commonly used approach in air pollution studies is spatial interpolation methods [29–31]. Spatially continuous data of environmental variables are often required for environmental sciences and management. However, information for environmental variables is usually collected by point sampling, particularly for the mountainous region and deep ocean area. Thus, methods generating such spatially continuous data by using point sampling become essential tools. Spatial interpolation methods are, however, often data-specific or even variable-specific. Many factors affect the predictive performance of the methods, and previous studies have shown that their effects are not consistent. Hence, it is difficult to select an appropriate method for a given dataset. Among many spatial interpolation approaches, a geostatistical method such as ordinary kriging (OK) is the most widely used method because they provide concentration estimates together with standard error at unmonitored locations [29].

Consequently, with increasing improvement in the analytical equipment and the development of new techniques, it is possible to detect a large number of air pollutants in ever-decreasing concentrations. On gathering information on air quality from monitoring stations, in spite of having a limited number of sampling points, it is possible to extract useful information using multivariate methods that are sufficient for the full description of a given scenario or situation.

Herein, the application of statistical methodologies such as multivariate analysis with principal component analysis (PCA) and cluster analysis (CA) or geostatistical analysis with ordinary kriging (OK) could be a very useful aid in their interpretation of air pollution data. So, in spite of the fact that Madrid and its suburbs constitute one of the largest metropolitan areas in Europe, fewer studies have been published regarding air quality in this area. Consequently, the aim of this work is to study and analyse the air quality of the city of Madrid (Spain), its metropolitan area, and the rest of the region on the basis of the levels of nitrogen oxides, ozone, and particulate matter (PM_10_), mapping the distribution of these pollutants and revealing the relationship between them and with the demography of the region. For this propose, correlation analysis, principal component analysis, cluster analysis, and geostatistical methodologies have been employed.

## 2. Materials and Methods

### 2.1. Sampling Points

**Figure 1:**
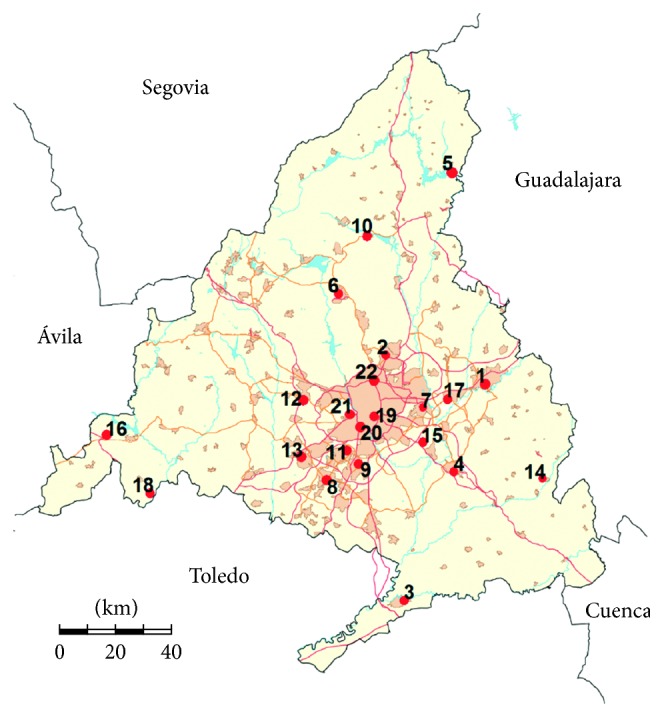
Locations of 22 monitoring stations in the Community of Madrid.

The Madrid Autonomous Region, officially called the Community of Madrid, is located in the centre of the Iberian Peninsula. Currently, there are more than 6.5 million people living in the region, of which nearly 85% live in the Madrid city and its metropolitan area, being largest in Spain, the 4th largest in the European Union, and the 54th largest in the world. The largest suburbs are in the south, and in general, along the main routes leading out of the Madrid city. Because of the pollution control policy, pollution levels have been decreasing in recent years [32] but it can once again worsen as a consequence of the end of the economic recession and the rise of fossil fuel consumption. The emission in Madrid region includes typical diffuse urban sources and industrial sources and traffic emissions due to the intense traffic across the major urban routes, ring road, and major highways crossing the region. For the control of the air quality, the Community of Madrid has two monitoring networks with air pollution control stations distributed along the region, one addressed directly by the Community government, formed by 23 automatic stations (13 urban, 4 suburban, and 6 rural stations), and the other network controlled by the municipal authorities of the city of Madrid, with another 24 stations (21 urban and 3 suburban stations). At these stations, pollutants such as SO_2_, VOCs, PM_10_, NO, NO_2_, CO, and O_3_ are continuously measured by reference methods. To date, only four of the pollutants NO, NO_2_, PM_10_, and O_3_ are simultaneously measured at 22 monitoring stations (Figure 1) being those the stations used for this study.

### 2.2. Air Pollution Data

Air quality data used in this study were collected in twenty-two stations of the Community of Madrid, eighteen of them corresponding to the network operating under regional authority control and the other four corresponding to the municipal air quality network of the city of Madrid. Pollutant concentrations were measured using reference methods [33]. Ozone with ultraviolet absorption at 253.7 nm, nitrogen oxides by means of the chemiluminescence analyzer, and PM_10_ was measured with a heated tapered oscillating microbalance (TEOM). The annual average concentrations in 2017 for NO, NO_2_, PM_10_, and O_3_ were calculated using the hourly and daily average concentration of the different air pollutants. The data corresponding to the city of Madrid were supplied by the Atmospheric Protection Service of the Madrid Council, available on its website [34] while those corresponding to the Madrid region are supplied by atmospheric quality area-air quality network of the autonomous region [35]. In both the cases, data are supplied in metadata text files that were transferred to Microsoft Excel sheet and then transformed in large datasets, compiled in tables where the relevant information was extracted and mathematically treated.

### 2.3. Multivariate Analysis

The statistical methods used in this study were Pearson's correlation coefficients, principal component analysis (PCA), and hierarchical cluster analysis (CA). The correlation coefficient is a statistic tool used to measure the extent of the relationship between variables, when compared in pairs. There are several types of correlation coefficients: Pearson's correlation is a correlation coefficient commonly used in linear regression and has been used to measure the strength of relationships between the four air pollutants. Principal component analysis (PCA) is a multivariate technique used to reduce the dimensionality of a dataset (preferably normally distributed, but not necessarily) [36] that contains a large number of interrelated variables, by transforming them into a new set of independent (uncorrelated) variables or principal components (PCs). When there is a significant correlation between variables, most of the variation in the dataset can be explained by considering a small number of principal components, and it becomes possible the visualization of patterns and correlations between the data while retaining as much as possible the information present in the original dataset. PCs are the eigenvectors of a covariance matrix or a correlation matrix, and each PC extracts a maximal share of the total variance. A PC with an eigenvalue greater or equal to 1 is considered as being of statistical significance (Kaiser criterion). Cluster analysis (CA) is a useful procedure used, as classification tool, very convenient in environmental studies because it simplifies and complements the PCA. Cluster analysis was undertaken following the Ward algorithmic method, which maximizes the variance between groups and minimizes it between members of the same group, and the Euclidean distance to compute the measurements. These statistical analyses were performed with the software package StatGraphics Centurion XVI 16.1.03, distributed by Statpoint Technologies, Inc., (Warrenton, Virginia).

### 2.4. Spatial Analysis

Spatial analysis or spatial statistics was performed to study topological, geometric, or geographic properties. It starts from the principle that there is a relationship between the distribution of analytes and that it is very likely that the values obtained from nearby points are more similar to each other than with values from more distant points. Spatial analysis includes a variety of techniques, many still in their early development, using different analytic approaches, such as spatial interpolation, also called Geostatistics which can be defined as the set of tools required to analyse spatial patterns and estimate the values of a continuous variable distributed in space or in time at unsampled locations. One of these methods is Ordinary Kriging, which uses only sample data for interpolation of the missing values and is considered as the best linear unbiased estimator (BLUE). Geostatistics tools applied to determine air pollution concentrations allow elaborating contour maps for a more visual assessment of the distribution of a certain pollutant. In this research, the contour maps of the four pollutants for the visualization of its spatial distribution were elaborated using ordinary kriging interpolation performed by means of OriginPro 8, (OriginLab Corporation, Northampton, Massachusetts, USA).

## 3. Results and Discussion

### 3.1. Data Description and Correlation Analysis

Table 1 shows the annual average concentrations recorded by the 22 monitoring stations selected for this study, together with their location and the affected population.

Descriptive statistics of the pollutants studied in the Community of Madrid have been carried out. A statistical summary of these data is shown in Table 2, including measures of central tendency, variability, and form.

As can be seen, the maximum annual average value obtained for NO_2_ is 62.9 *µ*g/m^3^, and it was registered by the monitoring station located at the very centre of Madrid. This station (E. Aguirre) together with four other stations of the metropolitan and nearby areas, Coslada, Getafe, and Leganés y Farolillo (stations 19, 7, 9, 11, and 20 in Table 1 and Figure 1), exceeded the annual average threshold for human health protection of 40 *µ*g/m^3^ established by the European and Spanish regulations [33, 38]. Regarding the particulate matter, PM_10_ has also an established annual average limit for human health protection of 40 *µ*g/m^3^ but none of the stations exceeded it. As for O_3_, all the stations taken into account registered values over 40 *µ*g/m^3^. Its established limit for human health protection is 120 *µ*g/m^3^ at the maximum mean of 8 hrs/day. Large standard deviations and coefficients of variation were found for the nitrogen oxides, which indicate heterogeneity in the concentrations among the monitoring stations. NO, NO_2_, and PM_10_ showed just slight deviation, while O_3_ was more skewed (asymmetry of the probability distribution) with a value of 2.3653, due to large differences between ozone measured levels in rural and highly populated areas.

Correlations analysis allowed to explore chemical/environmental associations among the studied pollutants which reflected the possible relationship between their sources. The results of the analysis are shown in Table 3. In all the cases, Pearson's correlation coefficients are significant between each pair of variables with *p* values ≤ 0.05. NO correlated significantly positively with NO_2_ (0.9442) and with PM_10_ (0.6314) that suggest a good association and a common source, despite the fact that, in Madrid, there are several episodes of Saharan dust intrusion throughout the year, a phenomenon relatively frequent in the Iberian Peninsula, which produces an increment of PM concentration in all regions in Spain [39].

The correlation coefficients of 0.6314 for the pair NO/PM_10_ and 0.5402 for NO_2_/PM_10_, although significant, suggest that the presence of the particulate matter in the Madrid region is mainly due to photochemical smog favored by the presence of nitrogen oxides, reinforced by the fact that the correlation with ozone is also significant. As can be seen, PM_10_ and O_3_ are also negatively correlated with a Pearson correlation of −0.7296.

### 3.2. Multivariate Analysis

#### 3.2.1. Principal Component Analysis

Principal component analysis was used in order to make possible the visualization of patterns and correlations between the data and hence the identification of possible emission sources. Eigenvalues and accumulated variance of the first two principal components are shown in Table 4.

**Figure 2:**
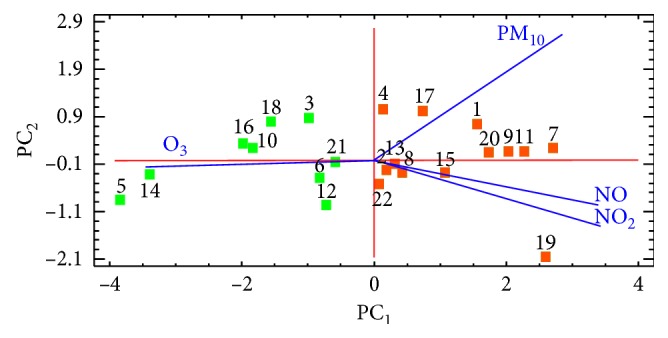
Biplot of scores and loadings for the first two principal components. Each point represents an air pollution monitoring station, and each line represents one of the variables.

Only principal components with eigenvalues higher than 1 should be retained (Kaiser criterion). In our data, according to this criterion, only the first component is enough to account for the 82% of the total variance, which is dominated by the loadings of NO, NO_2_, and O_3_ (loadings > 0.5) and can be associated with the origin of nitrogen oxides and their subsequent photochemical reaction to produce ozone from the road traffic. In order to facilitate the display of the data in two dimensions, the second principal component was also retained, which is mainly dominated by PM_10_ (loading > 0.8). This fact suggests some influence of other sources of particulate matter different from the photochemical reactions, probably with soil abrasion and/or episodes of Saharan intrusion. The biplot of scores and loadings for the first two principal components, accounting for 95% of the total variance, is presented in Figure 2.

This graph shows two different groups along the first principal component, which associates the incidence of each pollutant with their monitoring stations. The stations that have positive values of the first PC and therefore registered higher values of NO and NO_2_ (orange squares) are the stations located in densely populated urban areas, Madrid city and its metropolitan area, and close to main roads. On the other hand, the stations that have negative values of the first PC and follow the vector loading of O_3_ (green squares) are the stations located in sparsely populated rural areas and away from major roads. As can be seen, ozone pollution tends to be highest in rural areas away from the metropolitan area due to that certain pollutants that are more prevalent in urban areas, as nitrogen oxides, and they are present at lower levels of concentration.

#### 3.2.2. Cluster Analysis

**Figure 3:**
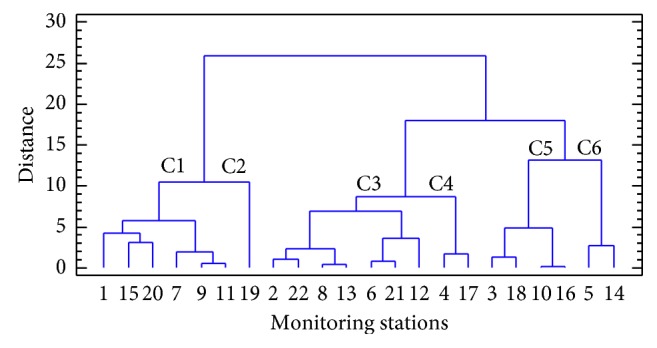
Dendrogram of the monitoring stations using Ward's method and Euclidean distance.

Cluster analysis (CA) is a useful multivariate method that was applied to find grouping patterns of pollution control station; each one of them described by variables such as nitrogen oxides, ozone, and particulate matter. Here, Euclidean distances and Ward's method were performed to measure the distances among the objects using as variables the annual average concentrations of the four studied variables for every station. The results of the CA are shown in the form of a dendrogram (Figure 3) that shows that the stations are classified into six major clusters.

Clusters 1 and 2 correspond to the stations situated in the southern Madrid city and in large towns in the Metropolitan area, located south and east of the city of Madrid while clusters 5 and 6 correspond to the stations situated in less-populated areas away from urban nuclei of the city in Madrid and in rural areas of the region. Finally, clusters 3 and 4 correspond to the suburban stations that surround the central area of the Community.

### 3.3. Geostatistical Analysis

**Figure 4:**
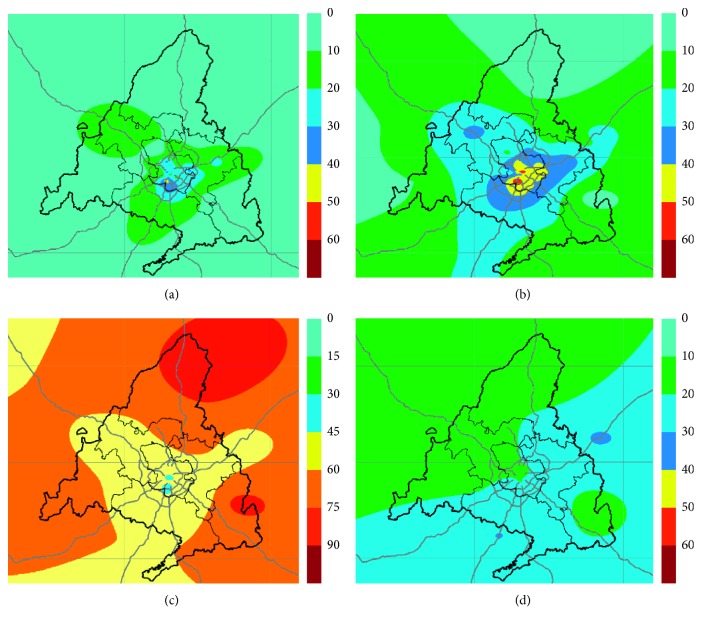
Contour maps of the spatial distribution of (a) NO (*µ*g/m^3^), (b) NO_2_ (*µ*g/m^3^), (c) O_3_ (*µ*g/m^3^), and (d) PM_10_ (*µ*g/m^3^) in the Community of Madrid in 2017.

Spatial interpolation method, ordinary kriging, was employed to find out if the data followed a normal distribution, as occured with NO, NO_2_, and PM_10_ which showed slight skewness as can be seen in Table 2.  Figure 4 shows the contour maps obtained by ordinary kriging interpolation.

The contour maps allow estimating the spatial distribution of the air pollutants in places where even no measurements of pollutants had been made. These maps show that nitrogen oxides are mostly concentrated in the east, west, south, and especially in the centre of the Madrid region, following the main roads and urban nuclei, where NO_2_ even exceeds the annual average limit for the protection of human health established as 40 *µ*g/m^3^ (yellow and red). The spatial distribution for PM_10_ shows that the most-affected areas are those in the south and east of the region but without exceeding the established annual average limit. In the O_3_ contour map, it can be seen how the rural zones away from large agglomerations are more affected by high levels of this pollutant (orange and brown).

**Figure 5:**
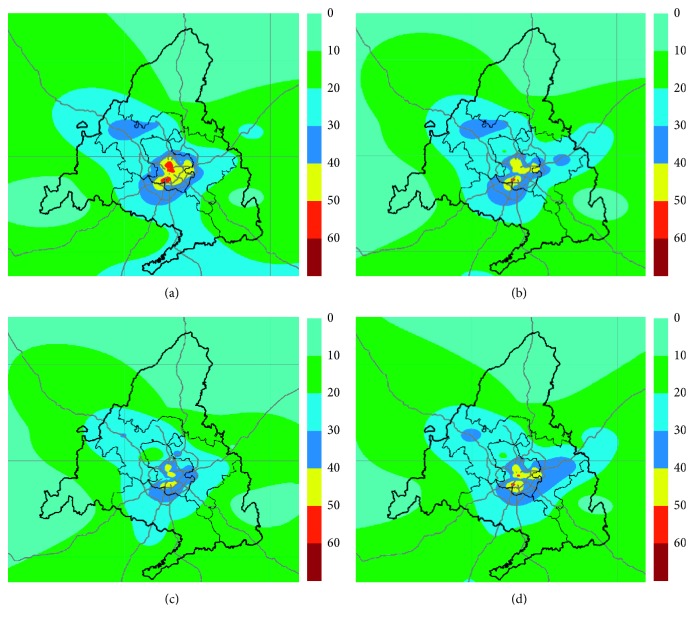
Contour maps of the spatial distribution of NO_2_ (*µ*g/m^3^) in the Community of Madrid for the years 2010, 2012, 2014, and 2016.

Finally, geostatistical analysis was used to study the evolution of the NO_2_ pollution level trend over the last few years. Contour maps for the years 2010, 2012, 2014, and 2016 have been provided (Figure 5).

A decreasing trend in the levels of NO_2_ has been observed in the period 2010–2014. This is the result of the air quality improvement plans implemented in the Community of Madrid [40, 41], focused mainly on the reduction of road traffic emissions. Another cause is related to the reduction of economic and industrial activities due to the economic crisis that began in 2008 [42]. The improvement of the economy and the rise of fossil fuel consumption since 2014 can be seen in the subsequent maps for 2016 and 2017 which show a slight increase in the NO_2_ levels in the metropolitan area and along the main roads.

## 4. Conclusions

The annual average concentration of NO, NO_2_, PM_10_, and O_3_ recorded by 22 stations in the network has been studied for a period from 2010 to 2017. An exploratory analysis shows that there is a correlation between these four selected pollutants. Principal component analysis (PCA) and hierarchical cluster analysis (CA) have allowed to establish the correlation between different variables (pollutants) and classify different monitoring stations, based on the significance of each variable, allowing to have a clearer view of the possible sources and mechanisms that govern air pollution. In addition, the ordinary kriging technique has been carried out to elaborate contour maps for the different pollutants. Thus, it has been possible to visually assess the spatial distribution of NO, NO_2_, PM_10_, and O_3_ in the Madrid region for 2017, which allows to establish the areas with the highest incidence and to see if the average annual limits established in the legislation for the different pollutants have been exceeded.

The contour maps for the period 2010–2017 using the ordinary kriging technique have reflected a decrease in the NO_2_ annual average concentration along the Madrid region from 2010 to 2014 and an increase from 2014 to 2017. Thus, it has been possible to establish a connection between this decrease and the measures proposed in the air quality improvement plans elaborated by the Community and the City of Madrid, since these are mainly focused on measures directly affecting the sources of this pollutant, that is, the traffic.

Hence, the results of PCA, CA, and ordinary kriging applied to air pollution data collected by the air quality monitoring networks have proved to be helpful in the assessment of the air pollution not only showing the incidence of four pollutants along the Madrid region but also providing a deeper insight into the major mechanisms involved.

## Figures and Tables

**Table 1:** Annual averages concentrations (*µ*g/m^3^) of NO, NO_2_, PM_10_, and O_3_ in the Community of Madrid.

Location number	Station location	Population^*∗*^	NO	NO_2_	PM_10_	O_3_
1	Alcalá de Henares	194310	23.6	37.0	26.5	53.3
2	Alcobendas	114864	17.1	32.3	21.1	57.4
3	Aranjuez	58213	5.1	16.4	22.2	60.4
4	Arganda del Rey	53821	9.0	24.0	24.1	52.7
5	El Atazar	97	1.1	5.2	14.6	85.2
6	Colmenar Viejo	48614	10.1	27.3	19.7	62.0
7	Coslada	83011	31.6	47.2	26.7	45.0
8	Fuenlabrada	194669	15.3	36.5	21.6	53.8
9	Getafe	178288	29.4	42.5	25.5	50.4
10	Guadalix de la Sierra	6049	3.6	12.4	19.1	65.3
11	Leganés	187720	29.4	43.1	25.4	46.2
12	Majadahonda	71299	9.6	30.2	17.3	56.3
13	Móstoles	206589	13.7	32.2	21.5	51.6
14	Orusco de Tajuña	1218	1.1	5.4	16.8	81.4
15	Rivas Vaciamadrid	83767	22.4	38.5	22.9	52.0
16	S. Martín de Valdeiglesias	8298	2.1	10.0	19.8	65.4
17	Torrejón de Ardoz	128013	12.8	30.7	25.4	51.4
18	Villa del Prado	6337	1.5	13.5	21.5	63.7
19	Madrid (E. Aguirre)	3182981	33.6	62.9	19.3	41.4
20	Madrid (Farolillo)	3182981	22.1	42.4	24.3	46.5
21	Madrid (Casa de Campo)	3182981	9.7	25.5	20.0	58.3
22	Madrid (Tres Olivos)	3182981	14.2	36.1	20.1	57.2

^*∗*^Official data corresponding to January 1, 2017 [37].

**Table 2:** Summary statistics of the air pollutants concentrations.

	NO	NO_2_	PM_10_	O_3_
Count	22	22	22	22
Average (*µ*g/m^3^)	14.5	29.6	21.6	57.1
Standard dev. (*µ*g/m^3^)	10.5	14.7	3.3	10.7
Coeff. of variation (%)	72.4	49.5	15.1	18.7
Minimum (*µ*g/m^3^)	1.1	5.2	14.6	41.4
Maximum (*µ*g/m^3^)	33.6	62.9	26.7	85.2
Range (*µ*g/m^3^)	32.5	57.7	12.1	43.7
Stnd. skewness	0.7919	0.0866	−0.3756	2.3653
Stnd. kurtosis	−0.9398	−0.1101	−0.4565	1.7539

**Table 3:** Pearson's correlation coefficients matrix among the four air pollutants (top). The sample size is given in brackets, and the *p* value is given in italic.

	NO	NO_2_	PM_10_	O_3_
NO	—	0.9442	0.6314	−0.8182
	—	(22)	(22)	(22)
	—	≤*0.0001*	*0.0016*	≤*0.0001*

NO_2_	0.9442	—	0.5404	−0.8928
	(22)	—	(22)	(22)
	≤*0.0001*	—	*0.0094*	≤*0.0001*

PM_10_	0.6314	0.5404	—	−0.7296
	(22)	(22)	—	(22)
	*0.0016*	*0.0094*	—	≤*0.0001*

O_3_	−0.8182	−0.8928	−0.7296	—
	(22)	(22)	(22)	—
	≤*0.0001*	≤*0.0001*	≤*0.0001*	—

**Table 4:** Eigenvalues and accumulated variance of the principal components.

Component number	Eigenvalue	Percent of variance	Cumulative percentage
1	3.295	82.4	82.3
2	0.525	13.1	95.5
3	0.165	4.1	99.6
4	0.014	0.4	100.0

## Data Availability

The data used to support this study were obtained from the Atmospheric Protection Service of the Madrid council and atmospheric quality area-air quality network of the Autonomous Region, and the data are available with an open access at the webpages of these departments. Details and references to the data have been given in Section 2.2.
